# An unusual cause of pulmonary artery aneurysm in an adult with repaired tetralogy of Fallot

**DOI:** 10.1093/ehjcr/ytag250

**Published:** 2026-04-16

**Authors:** Lais Costa Marques, Brianna Dryburgh, Felipe Soares Torres, Rafael Alonso-Gonzalez

**Affiliations:** Toronto Adult Congenital Heart Disease Program, Peter Munk Cardiac Centre, University Health Network, University of Toronto, Toronto General Hospital, 585 University Avenue, 5th floor, Toronto, Ontario M5G 2N2, Canada; Toronto Adult Congenital Heart Disease Program, Peter Munk Cardiac Centre, University Health Network, University of Toronto, Toronto General Hospital, 585 University Avenue, 5th floor, Toronto, Ontario M5G 2N2, Canada; Toronto Adult Congenital Heart Disease Program, Peter Munk Cardiac Centre, University Health Network, University of Toronto, Toronto General Hospital, 585 University Avenue, 5th floor, Toronto, Ontario M5G 2N2, Canada; Medical Imaging Department, University of Toronto, Toronto General Hospital, 585 University Avenue, 1st floor, Toronto, Ontario M5G 2N2, Canada; Toronto Adult Congenital Heart Disease Program, Peter Munk Cardiac Centre, University Health Network, University of Toronto, Toronto General Hospital, 585 University Avenue, 5th floor, Toronto, Ontario M5G 2N2, Canada

## Summary figure

(*A* and *B*) Chest X-ray: antero-posterior and lateral views showing left hilar opacity adjacent to a round opaque lesion (asterisk) and fibrotic changes in the left upper zone. (*C* and *D*) 3D volume rendered images: anterior oblique and cranial oblique (aortic arch removed) demonstrating the Pott’s shunt (arrow) and the left pulmonary artery aneurysm (asterisk). Axial non-ECG-gate computed tomography (*E*) demonstrating the Pott’s shunt (thin arrow), the left pulmonary artery (thick arrow), and the left pulmonary artery aneurysm (asterisk). Axial non-ECG-gate computed tomography post implantation of an occluder device in the Pott’s shunt (*F–H*) demonstrating lack of opacification of the aneurysm (asterisk).

**Figure:**
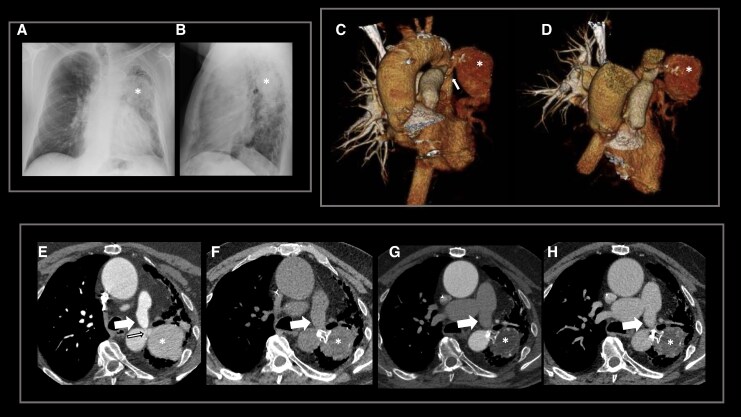


## Case description

A 44-year-old male with a history of tetralogy of Fallot (TOF) underwent palliation with a Potts shunt at 14 months, followed by complete surgical repair at age 13, including resection of infundibular stenosis, pulmonary valvotomy, ventricular septal defect closure, and Potts shunt ligation.

He was lost to follow-up until 2000, when he presented with NYHA class II symptoms. Chest X-ray revealed a round, opaque lesion in the left hemithorax, hypoplastic left lung, and cardiomegaly. Computed tomography (CT) imaging identified a large aneurysm of the left lower pulmonary artery (PA) measuring 45 × 49 × 58 mm, supplied by the Potts shunt, and complete occlusion of the left upper PA, likely ligated during the Fallot repair. Echocardiography showed mild left ventricular (LV) dilatation without valvar abnormalities.

We discussed the case in our multidisciplinary meeting and felt that the LV dilatation was likely related to his late repair rather than shunt-related volume overload. Conservative management with closed imaging surveillance of the aneurysm was recommended.

Unfortunately, no cross-sectional imaging was performed until 2022, when an updated CT showed progression of the aneurysm to 49 × 52 × 62 mm. A follow-up scan in 2023 revealed further enlargement to 48 × 55 × 64 mm. After evaluating both surgical and percutaneous options, the patient underwent successful percutaneous closure of the Potts shunt with a 16 mm Amplatzer device, with no acute complications.

Post-procedure, the patient experienced mild exertional dyspnoea, which resolved within 3 months. Follow-up CT demonstrated thrombosis of the aneurysm and reduction in its size.

## Supplementary Material

ytag250_Supplementary_Data

## Data Availability

There are no new data associated with this article. No new data were generated or analysed in support of this research.

